# Supplementary material for: Effect of exogenous alpha-tocopherol on physio-biochemical attributes and agronomic performance of lentil (Lens culinaris Medik.) under drought stress
Source: PLoS One. 2021 Aug 6;16(8):e0248200. doi: 10.1371/journal.pone.0248200 (PMC8345888; doi:10.1371/journal.pone.0248200)
Supplement: Attachment — Submitted filename: Response to the reviewer.docx [file pone.0248200.s001.docx]

| **Serial#** | **Comments by reviewer#1** | **Response by author** |
| --- | --- | --- |
| 1 | Highlights are missing. Add them substantially. | Highlights have been added accordingly. |
| 2 | Line 31: Revise it to “climatic conditions”. | The term “Climatic condition” is replaced with “climatic conditions” |
| 3 | Line 32: “ill” must be replaced with “drastic”. | The word “ill” is replaced with word “drastic”. |
| 4 | Line 33: Revise “induced drought” to “drought induced” | The word “induced drought” is revised to “drought induced” throughout the manuscript. |
| 5 | Line 34: Mention the abbreviation of “Alpha α” at the first place (line 32) and then use it throughout. | Instead of “alpha” abbreviation “α” is used throughout the manuscript. |
| 6 | Line 66: Add Comma after “findings” | Comma is added after word “findings”. |
| 7 | Line 74: Insert Comma after “plants” | Comma is added after word “plants”. |
| 8 | Line 79: Insert Comma after “regimes” | Comma is added after word “regimes”. |
| 9 | Line 83: Full stop after “Fabaceae” | Full stop is added after word “Fabaceae”. |
| 10 | Line 88: Change “induced drought stress” to “drought induced stress” in the whole manuscript. | “Induced drought” is revised to “drought induced” throughout the manuscript. |
| 11 | Line 113: Revise “potassium available” to “available potassium” | “Potassium (K) available” is revised to “available Potassium (K)” |
| 12 | Line 123: Please provide some detail about CVG in materials and methods. | Details of “CVG” added as suggested by reviewer#1. |
| 13 | Line 204: Seems like Alpha-tocopherol levels?? | “α” is added before tocopherol. |
| 14 | Line 205: Inset TAB at the start of the paragraph. | TAB is added at the start of paragraph. |
| 15 | Line 211: Insert comma after “treatments” | Comma is added after word “Treatments”. |
| 16 | Line 227: Add Full stop after “error”. Follow this for all the figures. | Full stop is added after word “error” throughout the manuscript for all figures |
| 17 | Line 239: Delete the word “group” after treatments. You have used this repeatedly. Please revise it carefully throughout as it is not making a good sentence structure. | Word “group” has been deleted after word “treatments”. |
| 18 | Line 250: It is better to mention all the figure legends together after the references. Please revise it. Follow this for all the legends. | All the figure legends are mentioned together below the references. |
| 19 | Line 262: Add comma after treatments. | Comma is added after word “treatments” |
| 20 | Line 266: Delete the word “condition” | The word “condition” has been removed. |
| 21 | Line 338: Add Comma after “condition” | Comma has been added after “condition”. |
| 22 | Line 338: “slow” must be replaced with “slows” | The word “slow” is correct according to grammar rules. |
| 23 | Line 342: Add comma after “study” | Comma is added after word “study”. |
| 24 | Line 343: Add comma after “contrary” | Comma is added after word “contrary”. |
| 25 | Line 350: Remove parenthesis after “in” | Parenthesis has been removed after word “in” |
| 26 | Line 354: Add Comma after “conditions” | Comma has been added after word “conditions” |
| 27 | Line 359: Add Comma after “study” | Comma has been added after word “study”. |
| 28 | Line 361: Add Comma after “contrast” | Comma has been added after word “contrast” |
| 29 | Line 362: Add Full-stop after figure number. | Full stop has been added after  (Fig. 4a-d). |
| 30 | Line 384: Add Comma after “condition” | Comma has been added after word “condition” |
| 31 | Line 387: Add parentheses (100 mg/L) | “100 mg/L” has been written inside parenthesis. |
| 32 | Line 392: Add Comma after “condition” | Comma has been added after word “condition” |
| 33 | Line 408: Add Comma after “results” | Comma has been added after word “results” |
| 34 | Line 421: Add “such as” after “enzymes” | Word “such as” is added after word “enzymes” |
| 35 | Line 423: Revise it “Our results are” | Word “were” is revised with word “are” |
| 36 | Line 426: Add Parentheses (200 mg/L). You did this mistake repeatedly. Please revise it throughout the manuscript. | Parenthesis have been added and revised throughout the manuscript. |
| 37 | Line 429: Add Comma after “study” | Comma has been added after word “study” |

| **Serial#** | **Comments by reviewer#2** | **Response by author** |
| --- | --- | --- |
| 1 | Simplify the title of the study. | Title has been simplified as “Effect of exogenous alpha-tocopherol on physio-biochemical attributes and agronomic performance of Lentil (*Lens culinaris* Medik*.*) Under drought stress” |
| 2 | Highlight the link between drought stress and oxidative burst, the potential mitigating effects and mechanisms underlying the mitigating effects of alpha-tocopherol. | A brief mechanism of mitigating effect of α–tocopherol under drought stress has been added in abstract section by highlighting the link between drought stress and oxidative burst. |
| 3 | In the abstract section, mention the strategy used for inducing drought stress. | The strategy used for inducing drought stress has been added in the abstract section. |
| 4 | An alternative word can be used for “Chaos” (L31). And throughout the manuscript, the term “environmental Chaos” could be avoided, as it seems quite subjective. | The term “Chaos” has been replaced with “adversities” throughout the manuscript. |
| 5 | The Introduction section can be improved by underlining the regional biophysical context to highlight how severe is drought stress in Punjab. | The required information has been added in the introduction section. |
| 6 | What were the reasons for selecting lentil as biological material in the present study? | Being rich in protein content, highly exportable to international markets and a major cash crop of Pakistan, were the main reasons behind selecting lentil as biological material in the present study. |
| 7 | There is also a need for more background information to justify selection of Alpha-tocopherol as alleviating agent against drought stress. | Further information has been added in the abstract, introduction and discussion sections. |
| 8 | The “Methods” part needs to be rewritten in a more rigorous way, by providing necessary information required for possible replication of the study. | The “Method” part has been improved, where improvement was needed. |
| 9 | What the ratio 2:1 stands for, since physico-chemical analyses suggested that the soil is sandy loam. Please clarify this. | The ratio of sand and silt was mistakenly written; it has been removed and replaced with accurate statement “with each pot containing 3kg sandy loam soil”. |
| 10 | What is the relative proportion of water and 70 % EtOH in the vehicule solution used for preparation of Alpha-tocopherol treatment? | The relative proportion of water and 70 % EtOH was 9:1 used for preparation of Alpha-tocopherol treatment. |
| 11 | What was the basis for selecting the different levels of Tocopherol treatments (100, 200, 300 mg/L) and those of drought treatments (20 and 25d drought periods). | Inferences from petri dish experiment revealed better responses in terms of radicles and plumules length with applied treatments of tocopherol used in pot experiment. Moreover, 20 and 25d drought periods were induced in the original trial because low levels of drought induced periods (5 to 15 d) did not show obvious physio-morphological effects on plant growth in the experiment that was designed |
| 12 | Please rewrite statement for statistical analyses, and integrate the approach used for correlation analysis. | Statistical analyses statement has been rewritten and the approach used for correlation analysis has also been added. |
| 13 | Please accurately present Dubois methodology for measurement of soluble sugar content. | Methodology for soluble sugar content has been rewritten and presented accurately. |
| 14 | The experiment was designed with two factors (tocopherol level, level of drought stress). However, authors appear to suggest three factors (L198). Please address this matter. | The experiment actually consisted of two factors, three factors were written mistakenly, and rectification has been incorporated in the revised manuscript. |
| 15 | The quality of graphs is rather poor… Please improve their resolution. And for better readability, the legend of different figures can be modified as (within drought stress period): (i) Control, (ii) Drought + 0 mg/L tocopherol, (iii) Drought + 100 mg/L tocopherol, (iv) Drought + 200 mg/L tocopherol, (v) Drought + 300 mg/L tocopherol. | Quality of graphs has been improved and the legends of graphs have been modified according to the suggestions given by reviewer#2. |
| 16 | Overall, the discussion didn’t help understanding the specific mode of action of Tocopherol in attenuating harmful effects of drought stress. Since drought stress is commonly related to stomatal closure, resulting in impaired Photosynthesis efficiency, it would have been interesting to clarify which parameter (s) in the photosynthesis apparatus was/were site(s) of Tocopherol beneficial action. | A detailed account of tocopherol, in attenuating harmful effects of drought stress and the site where tocopherol shows maximum activity has been mentioned in the discussion section from previous literature. |
| 17 | L303-306. This section is not at its appropriate place… Please check | Rectification has been done by arranging the small figures alphabetically within the main figure (figure #7). |
| 18 | Alternative expression can be found for “distinctly significant” | The term “distinctly” has been removed from results section, as it seems a bit inappropriate. |
| 19 | “Key metabolic activities”…What are they? | The key metabolic activities are photosynthesis and respiration. |
| 20 | Improve the conclusion section. | Conclusion section has been improved by adding logical arguments. |
| 21 | Add acronyms for each section. | All the acronyms in the manuscript have already been added below the abstract section. |
| 22 | How strong was the coefficient of correlation? | Correlation was significant at *P*≤0.05. The magnitudes of positivity and negativity of coefficient of correlation for different parameters have already been added in Table 2. |
