# Supplementary material for: Effect of exogenous alpha-tocopherol on physio-biochemical attributes and agronomic performance of lentil (Lens culinaris Medik.) under drought stress
Source: PLoS One. 2021 Aug 6;16(8):e0248200. doi: 10.1371/journal.pone.0248200 (PMC8345888; doi:10.1371/journal.pone.0248200)
Supplement: Attachment — Submitted filename: Response to the Reviewers.docx [file pone.0248200.s002.docx]

| **Serial#** | **Comments by reviewer#1** | **Response by author** |
| --- | --- | --- |
| 1 | Remove similar sentences from abstract and conclusion portion and make unique information for both these portions | Similar sentences have been removed and sentence structure has been transformed |
| 2 | Add highlights in bullet points. | Highlights have been changed to bullet points |
| 3 | Arrange the abbreviations on the first page to make them more obvious and precise | Abbreviations have been arranged on the first page. |

| **Serial#** | **Comments by reviewer#2** | **Response by author** |
| --- | --- | --- |
| 1 | Split the mentioned long sentences in shorter sentences and check the whole manuscript for the same flaw | Long sentences have been curtailed throughout the manuscript |
| 2 | Add highlights according to the journal instructions | Highlights have been written according to the journal format |

| **Serial#** | **Comments by editor** | **Response by author** |
| --- | --- | --- |
| 1 | Cross check the references and read manuscript for topographical and grammar errors | References cross checked and manuscript has been thoroughly read for topographical and grammar mistakes |
| 2 | Check references for retracted articles | References checked meticulously, no retracted articles were found |
